# From single molecules to life: microscopy at the nanoscale

**DOI:** 10.1007/s00216-016-9781-8

**Published:** 2016-09-09

**Authors:** Bartosz Turkowyd, David Virant, Ulrike Endesfelder

**Affiliations:** Department of Systems and Synthetic Microbiology, Max Planck Institute for Terrestrial Microbiology and LOEWE Center for Synthetic Microbiology (SYNMIKRO), Karl-von-Frisch-Str. 16, 35043 Marburg, Germany

**Keywords:** Super-resolution microscopy, Photophysics and photochemistry of fluorophores, Live cell imaging, Quantitative cell biology

## Abstract

Super-resolution microscopy is the term commonly given to fluorescence microscopy techniques with resolutions that are not limited by the diffraction of light. Since their conception a little over a decade ago, these techniques have quickly become the method of choice for many biologists studying structures and processes of single cells at the nanoscale. In this review, we present the three main approaches used to tackle the diffraction barrier of ∼200 nm: stimulated-emission depletion (STED) microscopy, structured illumination microscopy (SIM), and single-molecule localization microscopy (SMLM). We first present a theoretical overview of the techniques and underlying physics, followed by a practical guide to all of the facets involved in designing a super-resolution experiment, including an approachable explanation of the photochemistry involved, labeling methods available, and sample preparation procedures. Finally, we highlight some of the most exciting recent applications of and developments in these techniques, and discuss the outlook for this field.

**Graphical Abstract:**
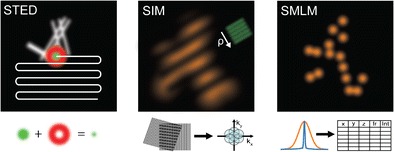
Super-resolution microscopy techniques. Working principles of the common approaches stimulated-emission depletion (STED) microscopy, structured illumination microscopy (SIM), and single-molecule localization microscopy (SMLM).

Super-resolution microscopy techniques. Working principles of the common approaches stimulated-emission depletion (STED) microscopy, structured illumination microscopy (SIM), and single-molecule localization microscopy (SMLM).

## Spatial and temporal scales in the life sciences and microscopy

**Fig. 1a–b:**
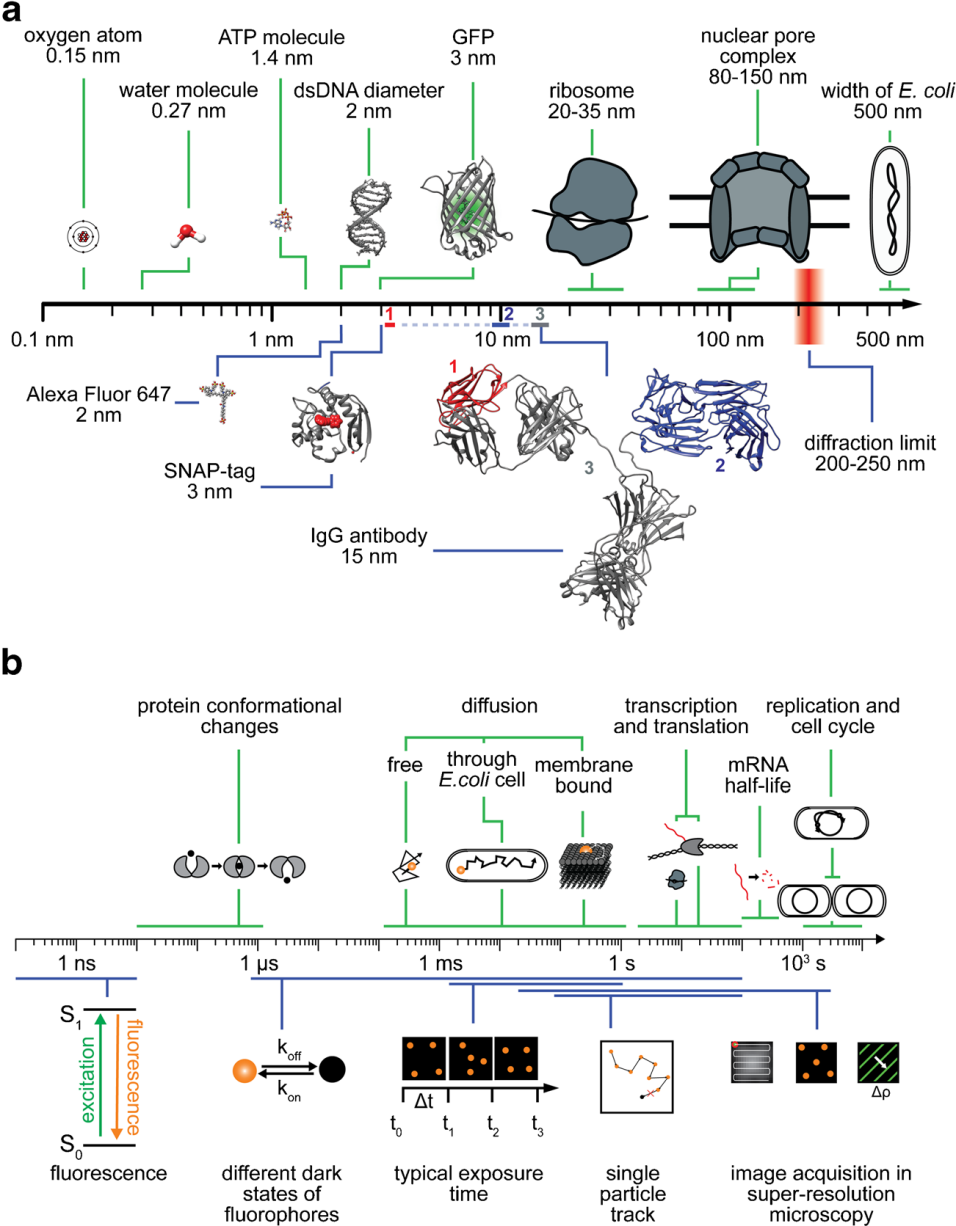
Spatial and temporal scales in the life sciences and microscopy. **a** Selected characteristic submicrometer objects are separated on the basis of biological (*above the axis*, *green*) and technical (*below the axis*, *blue*) significance. The IgG antibody structure (15 nm) contains two other notable structures: the antigen-binding region, called the Fab fragment (10 nm, *blue*) and the single-variable domain (3 nm, *red*), from which so-called nanobodies from cameloids are derived. Structures are taken from the PDB [GFP 1KYS, IgG 1IGT, SNAP 3KZZ, DNA 4LEY] and PubChem [ATP CID 5957, Alexa Fluor 647 CID 102227060]. **b** Timescales of various important biological processes (*above the axis*, *green*) and physical events, as well as typical timescales associated with microscopy procedures (*below the axis*, *blue*)

The timescales and spatial scales of the processes and molecules associated with life span extremely broad ranges, covering many orders of magnitude (Fig. 1). For instance, intracellular regulation (e.g., conformational changes or biochemical reactions within molecules) takes place at submillisecond timescales, nanosized molecules such as ATP (which serves the energy demands of cells) diffuse in milliseconds through cell volumes ranging from several micrometers up to millimeters, while (clustered) membrane receptors move at speeds that are about a magnitude slower. Large multicomponent machineries realize and control complex multilayered cellular functions that occur in seconds to hours. The ribosome, a large macromolecule which consists of two functional subunits of several dozen proteins on nucleic acid chain scaffolds, takes a matter of seconds to synthesize new peptide chains comprising hundreds of amino acids, which then quickly fold up into functional proteins. On the other hand, the replication of a full genome requires at least about 40 min for the 4.6 million nucleic acid base pairs of the bacterium *Escherichia coli*, and the cellular division cycle ranges from tens of minutes for *E. coli* to several hours for mammalian cells.

Observing and understanding all of these components of life requires us to be, at best, passive witnesses of undisturbed processes, but also to demand hard observational data that can allow us to quantitatively measure and trace all of the players involved—ranging from small molecules up to the interactions of whole cells in cellular communities—with the highest specificity and precision.

To achieve this, instrumentation is needed that permits a wide three-dimensional view but also allows details to be explored in high resolution, is noninvasive but can tell different cellular components apart, and offers detection that is rapid enough to be able to probe the processes of interest.

Today, the use of modern super-resolution fluorescence microscopes allows us to zoom into the intracellular structures of live cells [1, 2]. It is not only possible to resolve specimens in greater detail than naturally possible using the discriminating power of the human eye through the application of conventional light microscopes, but we are also able to circumvent the diffraction limit of light and study structures at near-molecular scale. This significant gain in resolution (which has revealed the heterogeneous nature of the lives of single cells), the inherent specific contrast of single fluorescent labels, and the ability to live-cell image single cells and large multicellular organisms have made fluorescence super-resolution microscopy one of the most powerful tools applied in the life sciences.

Nevertheless, there are limitations: the maximal photon flux of a fluorophore—which is mainly determined by its fluorescence lifetime—yields a lower bound for the detection range when observing molecular dynamics, and its maximum photon budget (above which it is irreversibly destroyed, i.e., photobleached) marks the upper bound for studying individual molecules. Technology-wise, minimal exposure times in the millisecond range limit the maximum observation rate of a planar live image array [3], and (for example) the sizes of labeling molecules such as dyes, protein tags, and antibodies yield steric resolution limits [4, 5]. Typical sizes of labeling molecules and the range of timescales of various life processes and imaging procedures are visualized in the lower panels of Fig. 1a and b.

**Fig. 2a–d:**
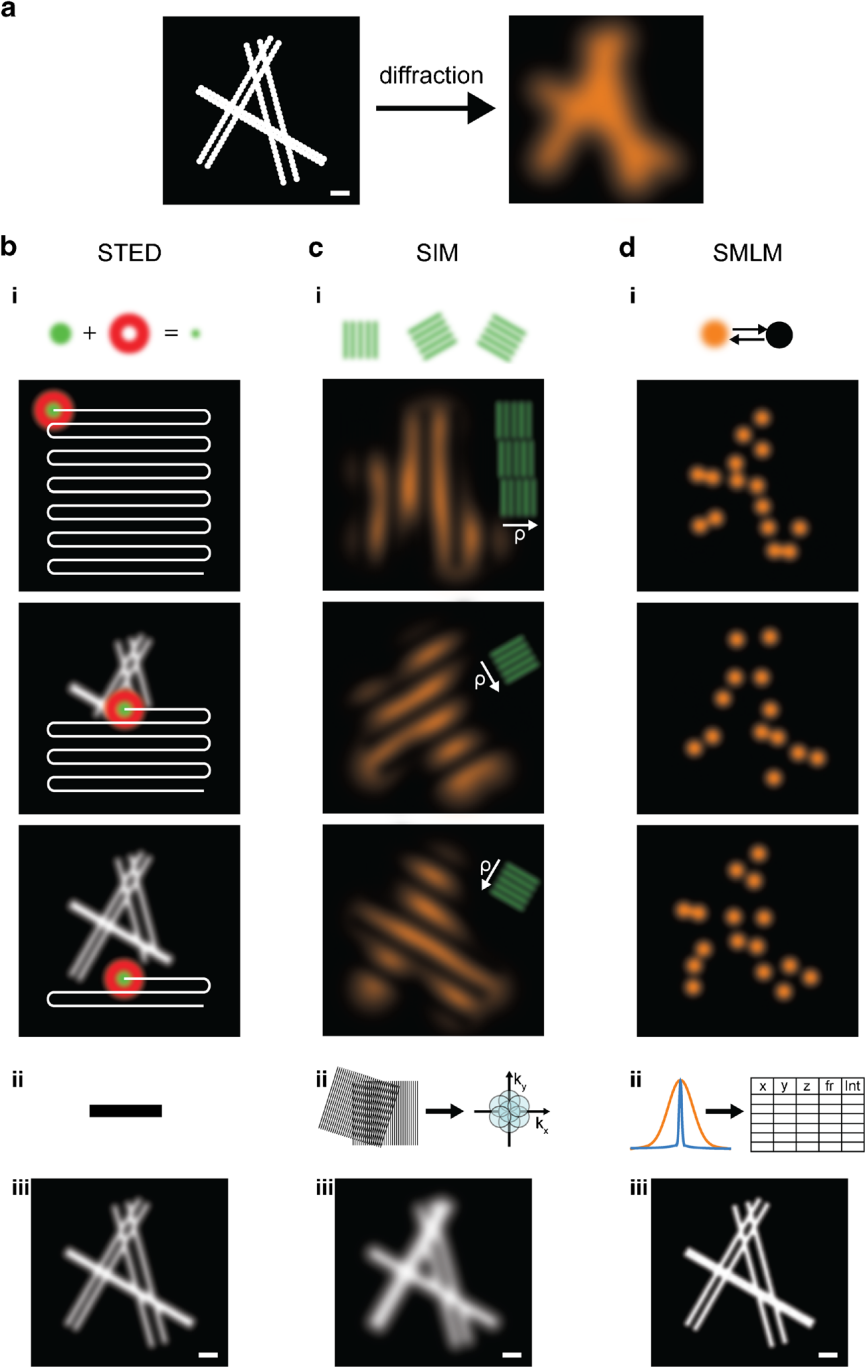
Principles of super-resolution microscopy techniques. **a** *Left*: Scheme of six filaments decorated with fluorophores (represented by large icons for visibility) and grouped into three pairs at simulated distances of 50, 100, and 150 nm;* scale* 200 nm.* Right*: A typical image of this structure obtained by conventional fluorescence microscopy is limited by the diffraction of light. **b** *i*, *top*: For STED, the structure is scanned by a subdiffraction excitation spot obtained by combining an excitation laser (*green*) with a, by phase-modulation shaped, depletion laser (*red*). After scanning the entire structure (*i*, *bottom*), and without performing any further post-processing steps (*ii*), an image is reconstructed (*iii*). **c** In SIM, fluorophores are excited by a series of regularly spaced illumination patterns of known frequency, orientation, and phase which modulate the fluorophore emissions. This results in visible low-frequency Moiré patterns that are dependent on the structure imaged (*i*). By analyzing the images for their spatial frequencies, an enlarged frequency space is obtained (*ii*), and a subdiffraction image is reconstructed (*iii*). **d** In SMLM, the fluorescence is modulated by photoswitching between “off” and “on” states. Most of the fluorophores are forced to reside in a dark off state; only a small subset of spatially separated fluorophores in the on state is allowed to emit fluorescence at a given time. After sequentially imaging thousands of subsets of fluorophores (*i*), the nanometer-precise fluorophore positions can be extracted from the diffraction-limited individual emissions (*ii*), and an image is reconstructed (*iii*). The three super-resolved images labeled (*iii*) visualize typical resolutions obtained by the methods: on the order of 50 nm (STED), 100 nm (SIM), and 20 nm (SMLM);* scale* 200 nm

It is important to point out that none of the advanced super-resolution microscopy techniques are routine methods as yet. They work close to current technological limits, and thus improve with each new implementation. Behind their stunning results and attractive images hide highly complex and tailored experimental designs. It is thus advisable to define the particular biological question to be answered as precisely as possible, and to plan biological experiments such that they suit the techniques well. Therefore, here, we will briefly review the basic principles of the three most widely used super-resolution microscopy techniques: stimulated-emission depletion (STED) microscopy [6], structured illumination microscopy (SIM) [7], and single-molecule localization microscopy (SMLM) [8–10], as depicted in Fig. 2 and summarized in Table 1. We explain, in detail, the essential characteristics of currently used reporter fluorophores, from their individual photophysics to general labeling strategies. Finally, we highlight the recent advances of the last few years, which have not only allowed the molecular compositions and structures of individual cellular components to be elucidated, but have also enabled us to place them into their native environmental context of large-scale spatial organization and to follow their dynamics. At the end of the paper, we emphasize the main challenges we currently face in order to achieve further improvements in these techniques and we introduce promising correlative schemes and sophisticated algorithmic and analytic tools which facilitate large-data and computational systems biology approaches.

**Table 1:** Overview of the characteristics of various super-resolution microscopy techniques

	STED	Linear SIM	Nonlinear SIM	SMLM
Microscope type	Laser scanning	Widefield	Widefield	Widefield
xy resolution (nm)	20–70	80–100	∼45	10–40
z resolution (nm)	30–100	∼300	∼170	10–50
Temporal resolution	ms to s	ms to s	ms to s	s to min
Laser intensities [W/cm^2^]	∼10^4^–10^9^	∼ 10–10^2^	∼ 10^2^–10^6^	∼ 10^3^–10^4^
Suitable fluorophores	Photostable fluorophores	All common fluorophores	Photostable or photoswitchable fluorophores	Photoswitchable fluorophores
Number of colors	3	3	1	4
Photobleaching	Moderate to high	Low to moderate	Moderate to high	Low to moderate (reversible switching) High (irreversible switching)

Values are taken from [10–15]

## Principles of super-resolution microscopy

The resolution of light microscopy is often introduced via the Rayleigh criterion. Light from point-like sources is convolved by the so-called point-spread function (PSF) of an optical system when transmitted through a diffraction-limited microscope (Fig. 2a). In 1896, Lord Rayleigh defined the maximum resolution of an optical system as the minimum distance between two point-like objects which can be separated as individual sources. He regarded two point sources of equal strength as just discernible when the main diffraction maximum of one image coincides with the first minimum of the other. For an epifluorescence microscope with a circular aperture where the light is collected with the same objective, this yields

$$ d=\frac{0.61\lambda }{\mathrm{NA}}, $$

where* λ* is the wavelength of light and NA is the numerical aperture [16].

Nevertheless, as already demonstrated by Zsigmondy using his ultramicroscope in 1902 [17], particles with dimensions below the resolution limit of visible light can be resolved. Also, confocal or multiphoton fluorescence approaches possess higher resolution than epifluorescence microscopes, as these techniques repress out-of-focus fluorescence, permitting straightforward three-dimensional imaging [18]. The resolution of near-field scanning microscopy (NSOM) is not limited by diffraction, as the diffraction limit applies only to light that has propagated a distance that is sufficiently larger than its wavelength. NSOM is therefore only limited by the aperture of the nanometer-sized excitation and detection tip placed near the sample [19].

Since the development of STED, the first far-field super-resolution fluorescence microscopy technique, many new methods that spatially or temporally confine fluorescence (which allows them to circumvent the diffraction barrier) have evolved. They can be categorized into two types of super-resolved far-field methods, with the first group concentrating on particular incident excitation light patterns and the second focusing on the modulation of the detected emission light over time. To be more specific, the first group, including techniques such as STED and SIM, make use of structured illumination schemes which spatially modulate the fluorescence of molecules such that not all of them simultaneously emit light. The second group, namely SMLM, rely on single-molecule imaging, and uses stochastic photomodulation of individual fluorophores. The number of photoswitchable fluorophores in their active fluorescent state can be controlled by irradiating the fluorophores with specific wavelengths of light. Thus, the stochastic activation of fluorescence at low rates allows the fluorescence emissions of single fluorophores to be spatially and temporally separated.

### Stimulated-emission depletion microscopy

In STED microscopy [6], the sample is scanned by a subdiffraction excitation spot. This spot is realized by superimposing two lasers: an excitation laser with a focused beam waist limited by diffraction and a STED depletion laser in a donut-shaped mode (achieved by phase modulation) with a wavelength at the far end of the fluorescence spectrum of the fluorophore used (Fig. 2bi). As a consequence, all of the fluorophores in the focal spot of the excitation laser are excited, whereas those located within the area of the donut-shaped STED laser are again quickly depleted from the excited state and forced back to their ground state by stimulated emission, resulting in the release of a photon identical to the incident STED depletion photon. This process only leaves fluorophores at the subdiffraction-sized central spot in the excited state, and their spontaneous fluorescence emission is measured. By precisely scanning the entire sample and measuring the respective fluorescence intensity of each subdiffraction area (Fig. 2bi, bottom), then, without the need for any further post-processing steps (Fig. 2bii), an image is reconstructed (Fig. 2biii).

The size of this effective subdiffraction scanning beam can be varied depending on the intensity of the STED depletion beam. The resulting resolution of STED microscopy can be described by

$$ {d}_{\mathrm{STED}}=\frac{d}{\sqrt{1+I/{I}_{\mathrm{s}}}}, $$

where *d* is the conventional resolution limit as defined by the Rayleigh criterion, *I* is the intensity of the STED depletion laser, and *I*_s_ is the effective saturation intensity, which can be defined as the intensity at which the probability of fluorescence emission is reduced by half [20].

By choosing the wavelength of the STED depletion laser to be at the far end of the fluorophore’s spectrum, reabsorption from the ground state as well as further absorption processes from the excited state can be neglected. Further, the resulting stimulated photons identical to the STED depletion wavelength possess a longer wavelength than the majority of photons obtained by spontaneous fluorescence emission. They can therefore be easily spectrally filtered, and—as stimulated emission also occurs on faster timescales—filtered temporally too. As it is a confocal technique, STED microscopy naturally permits optical sectioning, but three-dimensional imaging schemes have been further improved by, for example, creating an isotropic focal scanning spot using two opposing objectives [21].

The sample is scanned in steps as small as the effective subdiffraction-sized excitation spot, but is irradiated by the much larger, diffraction-limited, foci of the excitation and STED depletion lasers. Thus, the fluorophores are subjected to multiple excitation and de-excitation steps under high STED laser intensities, which requires them to be extraordinarily photostable. STED microscopy was initially realized in a pulsed laser scheme [6]; continuous-wave illumination STED microscopy was implemented later [22]. However, rather high intensities are required in both imaging schemes, leading to increased photobleaching and phototoxicity in the sample. This negative effect can be reduced by employing sophisticated imaging modes which lower or shorten the applied laser intensities, e.g., by time gating [23], by selective, feedbacked use of the depletion beam to reduce the number of state transition cycles [24], or by replacing (a concept also termed RESOLFT: reversible saturable optical (fluorescence) transition) [25] or assisting [26] the stimulated depletion mechanism with an on–off photoswitch. STED utilizing moderate laser power schemes can be applied to the imaging of live cells as well as living tissue and living organisms (for a detailed review, see [27]).

For multicolor STED microscopy, either a pair of lasers is required for each fluorophore [28], or, for spectrally close fluorescence spectra, only one depletion laser is needed [29]. This further automatically coaligns the effective scanning spots of both colors. Very specific fluorophore pairs, chosen to be suitable for spectral demixing approaches or to demonstrate reverse photochromic behavior, can be operated by just one pair of lasers [30, 31].

### Structured illumination microscopy

SIM uses regularly spaced patterns of known spatial frequency, orientation, and phase to illuminate the sample by a structured excitation light series ([7]; detailed review [32]). This leads to modulated fluorescence emissions which form defined interference Moiré fringes of high and low frequencies, as the light emitted from a specific point in the sample is the product of the local structure of the sample (more precisely the spatial distribution of the fluorophores) and the local excitation intensity. As the corresponding fluorescence image seen through the microscope is diffraction limited and thus convolved with the PSF of the optical system, only the low-frequency Moiré patterns can be measured. These structure-specific patterns are registered for different sequential phases and orientations of the illumination pattern to sample the maximum isotropic frequency space (Fig. 2ci). By measuring the apparent Moiré fringes and knowing the properties of the chosen illumination patterns, it is possible to retrieve information at higher spatial frequencies than normally possible in a widefield microscope (Fig. 2cii): the diffraction limit can be described as a circular boundary in the transmitted frequency space with a maximal frequency of* k*_max_, equaling 1/*d*. Thus, only the spatial frequencies with* k* ≪  *k*_max_ pass through the optical system. Using structured illumination, which allows the detection of low-frequency Moiré interference patterns, spatial information about the sample from higher frequency bands is shifted into detectable lower frequency bands. All of the acquired images can be analyzed for their spatial frequencies and then be unmixed by their multiple overlapping components in frequency space. This allows the high frequencies obtained using the Moiré information to be shifted back to their original frequencies. The resulting enlarged frequency space encompasses about 2*k*_max_, as the low-frequency Moiré patterns must remain visible above the diffraction limit. Using an inverse Fourier transform back into image space, a super-resolved SIM image showing a linear twofold increase in resolution can then be reconstructed (Fig. 2ciii).

Ignoring for a brief moment the rather complex post-processing of the raw data acquired by sophisticated SIM software (recently published open-source options are [33, 34]), SIM is the most straightforward approach in the field of super-resolution microscopy: the technique is based on standard widefield fluorescence microscopes, only requires (in the simplest version of SIM) a movable grating placed in a Fourier plane of the illumination path, and works for all common (albeit best for bright) fluorophores. SIM can be used to image live cells [11, 35] and has been extended to three-dimensional SIM [12], is capable of imaging live organisms [36], and allows for multicolor imaging [37]. Nevertheless, common artifacts (arising from imperfect imaging or algorithms) should be carefully considered, avoided, or corrected for: stripes in a reconstituted SIM image emerge from photobleaching, sample drift, or setup vibrations, a low fluorescence modulation contrast results in noise in the high-frequency range, and spherical aberration as well as refractive index mismatching creates halos or the doubling of features [38].

A higher resolution than that obtained by linear SIM is achieved by nonlinear SIM, which is realized by either saturating the fluorescence through the application of strong illumination intensities [39] or by using photoswitchable fluorophores [13, 40] (similar to RESOLFT [25]) to create illumination patterns that include higher harmonic frequencies. However, the increased resolution of this technique comes at the expense of a limited choice of fluorophores, which need to be either highly photostable (in order to withstand the strong illumination intensities) or photoswitchable. The resolution obtained using SIM approaches can be determined via

$$ {d}_{SIM}\approx \frac{d}{2+h}, $$

where *d* is the conventional resolution limit and *h* is the number of higher harmonics achieved when applying nonlinear SIM schemes [13, 39, 40]. For linear SIM, *h* equals zero, so the resolution enhancement is about twofold.

### Single-molecule localization microscopy

Single-molecule localization-based techniques such as photoactivated localization microscopy (PALM) ([8], (*direct*) stochastic optical reconstruction microscopy ((*d*)STORM) [9, 10], ground-state depletion followed by individual molecule return (GSDIM) [41], and many other related techniques [42] are commonly grouped together under the term “single-molecule localization microscopy” (SMLM). They all require tight control over the photoswitching of individual fluorophores, as discussed in detail in this review, and they rely on the use of post-processing algorithms to generate the super-resolved data (see the review by Small and Stahlheber [43] and comparative studies of localization algorithms [44] and single-particle tracking algorithms [45]; most of the relevant algorithms are openly available).

In SMLM, the main principle is stochastic photoswitching and the detection of single spatially separated fluorophores. To achieve this, all fluorophores are modulated by photoswitching them between “off” and “on” states. Most of the fluorophores are forced to reside in a long-lasting dark off-state; only a small subset of fluorophores in the on state are allowed to emit fluorescence at a given time. By sequentially imaging typically several thousand subsets of spatially distinguishable fluorophores, all of the emitters are detected over time (Fig. 2di). The photons emitted from the fluorophores are distributed in diffraction-limited spots and registered in a stack of time-resolved images until all of the fluorophores have been read out. The spots can be identified by image-processing algorithms, allowing the positions of the fluorophores and other properties (fluorescence intensity, duration of fluorescence, precision of the positioning fit, etc.) to be precisely determined and then stored in a large table (Fig. 2dii). Using the fluorophore centroids, a super-resolved image is reconstructed (Fig. 2diii).

The resolution limit of SMLM is mainly determined by the precision with which individual fluorophores are localized, which can be simplified to

$$ {d}_{\mathrm{SMLM}}\approx \frac{d}{\sqrt{\mathrm{N}}}, $$

where *d* is the conventional resolution limit and *N* is the number of photons detected in a single fluorescence spot [46].

SMLM approaches are more sensitive to background signals than both of the previously described methods, as SMLM determines the positions of individual molecules to a high precision based on their individual fluorescence levels. To assign as many photons as possible to a single fluorophore, it is highly desirable to achieve the best possible signal-to-noise ratio. For thin (mainly two-dimensional) samples, effective background noise reduction can be achieved using total internal reflection fluorescence microscopy (TIRF), where the incident laser light is totally internally reflected at the glass–water boundary between the coverslip and sample [47]. In this illumination scheme, only the fluorophores in a very thin layer within the exponentially decaying evanescent field above the coverslip can fluoresce. Thus, a large fraction of the usual background signal caused by autofluorescence or by the scattering of the laser light and originating from the whole sample volume is suppressed. Another approach is to illuminate the sample in a highly inclined and laminated optical sheet (HILO) [48]. In this mode, the excitation laser light leaves the objective at a very narrow angle, which results in an inclined beam passing through the sample. This illumination in the form of an optical light sheet is then almost perpendicular to the detection path of the microscope.

To allow for three-dimensional SMLM imaging, several optical methods have been utilized to encode the third dimension: astigmatic PSF shaping by a cylindrical lens, biplane alignment, a dual-objective scheme allowing for the interference of the signal, and several further phase modulations have been developed that (for example) create a double-helically arranged PSF or a self-bending PSF which spans a large field of view at isotropic resolution [1]. These three-dimensional SMLM read-out schemes can be combined with spatially confined activation approaches based on temporal focusing [49], selective plane illumination microscopy [50], or lattice light sheet illumination [51].

SMLM allows for multicolor imaging if the photoswitching mechanisms of the fluorophores used fit together well; i.e., when they tolerate the same imaging environment such as the same specialized switching buffers [52–54] or a mounting medium combined with high laser intensities [55], by employing complementary photoactivation schemes [56, 57], or by using dye activator–reporter pairs [58]. Most multicolor approaches are assisted by sophisticated read-out schemes [59–63]. We discuss how to choose appropriate fluorophores to use in a particular study and the parameters that should be taken into account in the next section of this review, where we introduce the basic photophysics and explain how to switch or stabilize fluorophores.

Structural live-cell SMLM imaging of only slowly changing structures can be performed as the imaging speed is fast compared to the phenomenon being imaged. For these structures, it is possible to capture a sufficient number of subsets of fluorophores to fill a subdiffraction sampling space before the structure has changed significantly. Nevertheless, a gain in temporal resolution will always result in a loss of structural spatial resolution caused by lower sampling, and vice versa [55, 64–66]. Uniquely, SMLM can be combined with single-particle tracking (SPT); unlike diffraction-limited SPT methods, where only a strictly limited number of fluorophores can be followed per cell to keep them separable, sptPALM [67] is readily capable of measuring a large batch of statistics on single-molecule tracks for the same type of molecule inside a single cell by sequential photoactivation. It is thus possible to obtain spatially and temporally highly resolved diffusion maps that combine a multitude of tracks and accordingly unravel possible dynamic heterogeneities and subpopulations. sptPALM has been applied to a wide range of biological systems (some examples are given in [68–70]), and can be combined with structural SMLM imaging [71]. It is nevertheless important to note that the minimum time needed to precisely localize a single fluorophore is influenced by imaging parameters such as the camera sensitivity, the minimum applicable acquisition times (in the range of a few milliseconds), as well as the contrast of the fluorophore (determined by its quantum yield in the specific sample, the laser intensities, and the background noise). This means that sptPALM is only well suited to studying slow diffusion processes, where the fluorophores move slowly compared to the image acquisition time; it is not applicable to processes with faster dynamics such as that visualized in Fig. 1b.

## Designing the optimal experiment

### Choosing a suitable fluorophore

**Fig. 3a–b:**
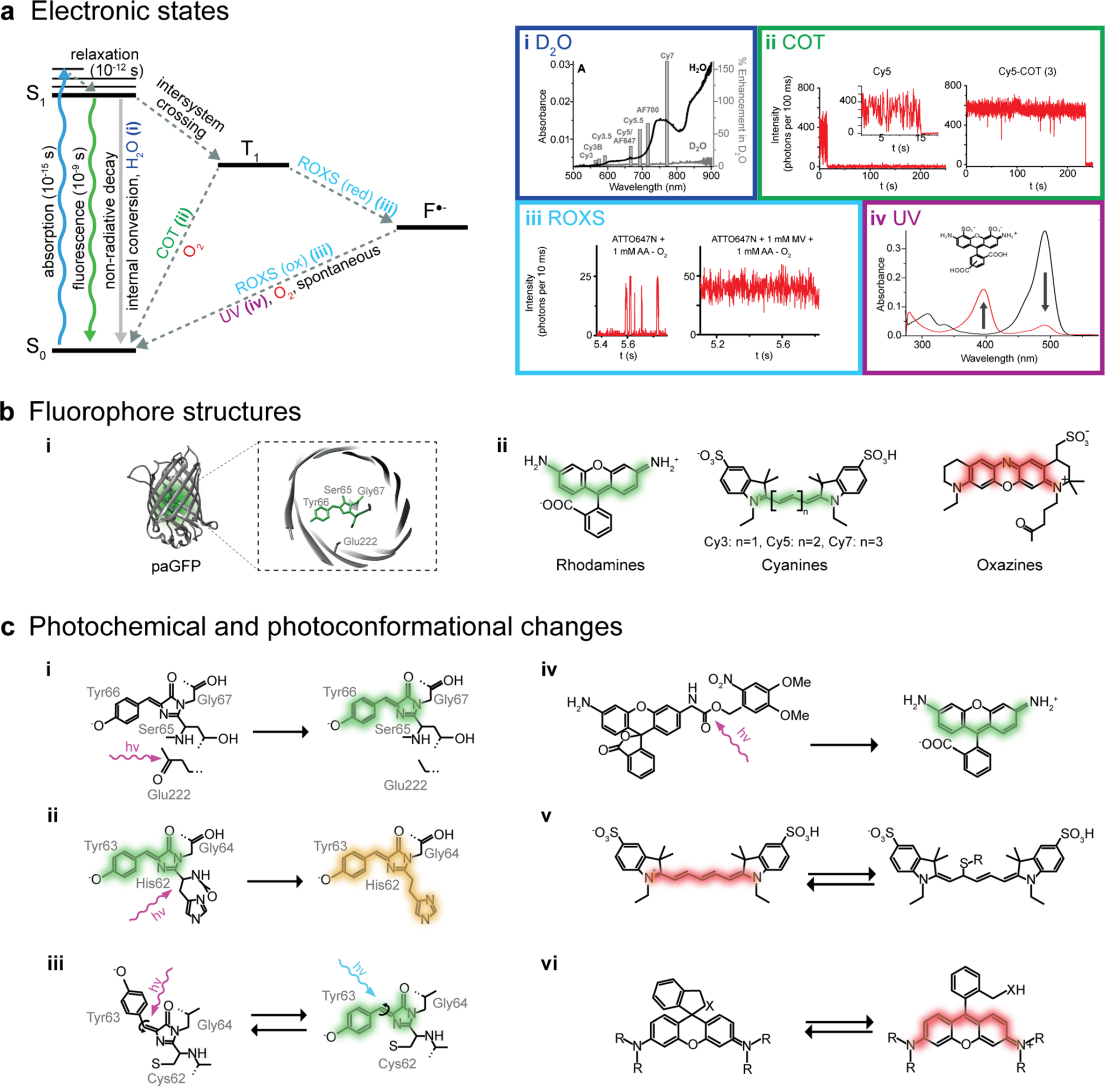
Photophysics and photochemistry of fluorophores. **a** *Left*: Jabłoński energy diagram representing energy states and transitions of a fluorophore.* S*_*0*_ ground singlet state,* S*_*1*_ excited singlet state,* T*_*1*_ triplet state,* F* ^●−^ radical state. Different compounds can affect brightness and photostability or shift the fluorophore into a radical state. (*i*) absorption spectra of H_2_O and D_2_O, and correlated enhancements of the fluorescence emissions of different fluorophores in D_2_O versus H_2_O for the visible range of light. Adapted from [72] with permission. (*ii*) Cyclooctatetraene (COT) quenches the triplet state by quickly transferring fluorophores back into the ground state and thus stabilizes the fluorescence. Adapted with permission from [73]. (*iii*) A reducing and oxidizing system (ROXS) accelerates the transition of a fluorophore from its triplet state back to the electronic ground state by performing fast sequential reducing and oxidizing steps. Adapted with permission from [74]. (*iv*) The radical states of some dyes (e.g., the Alexa Fluor 488 fluorophore, as shown in *black* here;* red* indicates the radical) possess an absorption peak in the UV range. By exciting the radicals with UV light to higher intermediate states, they can be quickly brought back down to their electronic ground state. Adapted with permission from [75]. **b** Different fluorophore structures: (*i*) Barrel structure of the photoactivatable green fluorescent protein (paGFP) and a close-up of its chromophore. (*ii*) Overview of organic dye classes. **c** Different photochemical and conformational changes that affect fluorescence: (*i*) photoactivation of paGFP [76], (*ii*) green-to-red photoconversion of mEos2 [77], (*iii*) reversible* cis*/*trans*-photoswitching of Dronpa [78], (*iv*) cleavage of a photocage from a rhodamine [79], (*v*) reversible fluorescence quenching of Cy5 by covalent binding of a thiol [80], and (*vi*) reversible cyclization of rhodamine HMSiR [81]

Normally, fluorophores reside in their most relaxed molecular state, the electronic ground state (S_0_). When a fluorophore absorbs a photon, it is excited within femtoseconds to a higher energy state (S_1_, S_2_, …, S_*n*_). Depending on the exact energy of the absorbed photon, the fluorophore can be excited to various energy levels that correspond to its electronic, vibrational, and rotational molecular configurations. As depicted in Fig. 3a (in which, for simplicity, only the S_0_, S_1_, and T_1_ electronic states and the vibrational states for S_1_ are shown), the fluorophore then relaxes within picoseconds to the lowest level of the excited electronic state S_1_, transferring its vibrational energy to its surroundings.

The time a fluorophore spends in the lowest level of the excited state, often called the fluorescence lifetime, is normally in the nanosecond range, though it depends on the specific molecule and its environment. When returning to the electronic ground state, nonfluorescing molecules release their energy through nonradiative processes such as internal conversion. Fluorophores, on the other hand, exhibit a high probability of a radiative transition; they release energy through the emission of a single fluorescence photon. As a portion of the energy is also lost before this transition through vibrational state relaxation, the fluorescence photon actually has a longer wavelength than the wavelength of the photon originally absorbed. This phenomenon is known as the Stokes shift.

Besides relaxing directly to the ground state through either photon emission or nonradiative internal conversion, an excited fluorophore can release its excess energy by undergoing several other intramolecular and intermolecular processes. Such events decrease the photon yield and are collectively termed quenching. Through the intramolecular transition process known as intersystem crossing, fluorophores can reach an intermediate energy state called the triplet state (T_1_). This process involves flipping the spin of the excited electron, and has a miniscule probability (i.e., it is quantum-mechanically forbidden) of occurring during each excitation–relaxation cycle. The triplet state has a much longer lifetime, typically several microseconds, during which the excited molecule remains prone to electron transfer reactions. The result of such a reaction can be a nonfluorescent radical state (F^●−^) in which the fluorophore can remain for several seconds or even minutes. Occasionally it can result in irreversible destruction of the fluorophore through photobleaching processes, leading to a permanent loss of fluorescence. Other dark, nonfluorescent states can be caused by conformational changes in the chromophore, the formation of complexes with other molecules, or a collision with a molecule that is capable of receiving the fluorophore’s surplus energy (e.g., oxygen, halogens, and amines). Collisional quenching requires direct proximity of the quencher molecule to the chromophore, and its rate is drastically decreased in fluorescent proteins where the chromophore is protected by its beta-sheet barrel (Fig. 3bi). Finally, the energy of an excited fluorophore can also be transferred to another molecule by photoinduced electron transfer (PET) or Förster resonance energy transfer (FRET), both of which are often exploited in advanced imaging schemes that measure interaction dynamics within or between proteins of interest [82].

The most common types of fluorophores are fluorescent proteins such as GFP (Fig. 3bi) and organic dyes such as rhodamines, carbocyanines, and oxazines (Fig. 3bii). At the heart of every fluorophore is the chromophore, a conjugated π-electron system that gives a molecule its light-absorbing properties. A chromophore can consist of aromatic rings as well as C=C, C=O, or N=N bonds. Its spectral properties are determined by the length of the conjugated electronic system, the number of electrons, and different substituents [83]. Usually, elongation of the conjugated system will shift the absorption maximum and thus also the emission maximum to longer wavelengths. This can easily be seen in cyanines, a class of fluorescent dyes with different polymethine chain lengths. Stretching the chain from Cy3 to Cy7 shifts the emission spectrum from green to dark red (Fig. 3bii). Every fluorophore thus possesses a unique excitation and emission spectrum. These spectra need to be compatible with the available microscopic system (i.e., in terms of illumination wavelengths, spectral filter combinations, or the sensitivity of the given detector). The excitation and emission wavelengths should be separated by a sufficiently large Stokes shift, and, in multicolor experiments, the chosen set of fluorophores should exhibit sharp and defined spectra with ideally no overlap, thus minimizing crosstalk between the different colors. Alternatively, overlapping spectra can be separated by spectral demixing approaches, which also nicely avoid chromatic aberrations and can allow the use of a single excitation source [60–63]. Sample specifications must also be taken into account; live cells are usually more sensitive to irradiation with shorter-wavelength light; imaging for extended periods of time with light in the ultraviolet (UV) range can lead to a range of defects in cells, from DNA damage to death [84]. Certain biological samples exhibit pronounced autofluorescence in some spectral ranges, usually in shorter wavelengths. The majority of this background fluorescence is caused by aromatic amino acids (mainly tryptophan), the phosphate chain of DNA, intracellular nicotinamide adenine dinucleotide (NADH), and coenzymes [85]. Longer wavelengths of light can penetrate deeper into a tissue, making red and near-infrared fluorophores the most suitable for imaging thicker samples [18].

The chosen fluorophore should be as bright as possible to ensure that sufficient signal is detected to allow it to be distinguished from the background. This is especially crucial for single-molecule imaging when the fluorescence of individual fluorophores is captured. The fluorophore’s brightness is determined by its dipole orientation in relation to the excitation light, its extinction coefficient (which quantifies how well a fluorophore absorbs a certain wavelength), and its quantum yield (the ratio of absorbed to emitted photons). Ideally, the excited fluorophore would emit a single photon in every excitation–emission cycle, thus exhibiting a quantum yield of 1. However, due to the alternative process of excited-state relaxation described earlier, this is not the case in practice. A fluorophore featuring a relatively low quantum yield can nevertheless produce a sufficient fluorescent signal, provided that its extinction coefficient is high enough and its rate of entry into the excited state is maximized by applying high excitation light intensities, leading to more rapid cycling through the excitation–emission cycle (Fig. 3a).

A constant flux of emitted photons (i.e., the fluorophore’s photostability) is another important factor. Fluctuations in fluorescence can be attributed to reversible or irreversible losses of fluorescence, and depend on the chemical properties of the fluorophore, its environment, and the light intensities that it is exposed to. Oxygen and reactive oxygen species play a large role in irreversible bleaching, which is caused by a permanent change in the molecular structure of the fluorophore [86]. Absorption of a second photon while already in the excited state is believed to be another major cause of photobleaching. Low irreversible bleaching rates allow for longer measurements or at higher excitation light intensities. Reversible losses of fluorescence are caused by transitions to several intermediate nonfluorescent electronic or conformational states, as sketched in Fig. 3a. Minimizing the time a fluorophore spends in these states improves the fluorescence signal stability and increases the time that a fluorophore spends performing its excitation–emission cycle, yielding a more constant photon flux.

The solubility and cell permeability of fluorophores must also be considered. Relatively few fluorophores can be transported through a live cell membrane (these are highlighted in Table 2) due to either size or charge constraints. Fluorescent proteins are highly live-cell compatible but can be a steric hindrance in some cases, and can impact cell viability when fused to certain proteins. They have also been shown to form artificial aggregates, depending on the abundance and spatial organization of the target molecule [122].

**Table 2:**
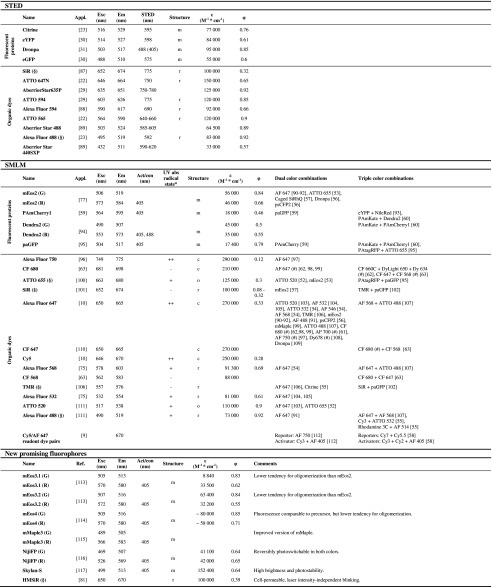
List of recommended and promising new fluorophores for super-resolution microscopy

Numbers are taken from the manufacturers and from [59, 77, 81, 94, 101, 113–120]

*Appl* Example of the use of the fluorophore for super-resolution microscopy, *Exc* excitation peak, *Em* emission peak, *Act/con* activation or conversion wavelength, *ε* molar extinction, *φ* quantum yield, *m* monomeric, *c* cyanine, *o* oxazine, *r* rhodamine, (§) cell-permeable dyes

* [75, 121]; (#) spectral demixing

Importantly, each super-resolution technique has special demands. The most common fluorophores employed, their properties, and (in the case of SMLM) the most popular multi-color combinations are given in Table 2. In STED, the molecules are constantly forced from the excited state into the electronic ground state via stimulated emission. Fluorophores with high extinction coefficients, high quantum yields, and high stimulated emission cross-sections are favorable, as they allow for the best possible contrast in the detection of the fluorescing fluorophores left in the center of the excitation pattern. The rate of stimulated depletion of the excited state scales with the depletion energy applied, so fluorophores chosen for STED have to be exceptionally photostable. Further, the depletion wavelength should be carefully chosen to ensure that it does not re-excite any of the fluorophores that are depleted to the ground state. For SIM, the most crucial parameters are the photostability and overall brightness of the fluorophore, as the technique works by measuring the fluorescence response of a defined patterned excitation. This modulation of fluorescence should be clearly detectable based on a strong and inherently stable fluorescence signal. Since illumination-independent fluctuations in fluorescence result in artifacts, the use of an effective antifading agent is common practice. Almost all modern fluorophores can be used for SIM (which is why we do not provide a selection of SIM fluorophores in Table 2). Finally, for SMLM techniques, rigid control of photoswitching is crucial. The nonfluorescing dark times of the fluorophores must be long enough to guarantee the separation of single-molecule signals in the sample at any time during the experiment. Even when applying algorithms that can handle high numbers of fluorescent molecules at a time, the techniques are easily impaired when the density of molecules is too high [123].

The dye Alexa Fluor 647 is the fluorophore of choice in a great majority of fixed cell SMLM studies, due to its robust photoswitching and good photon yields. Since it is not membrane permeable, ATTO 655, tetramethylrhodamine, SiR, and Oregon Green are utilized in most live cell studies. When multicolor imaging is desired, Alexa Fluor 568 and 532 are often used with Alexa Fluor 647. Fluorescent proteins are more suitable for quantitative approaches or noninvasive live cell studies. A collection of popular fluorophores as well as multicolor schemes is provided in Table 2. In this context, different photoswitching strategies (as evaluated in detail below) require individual optimizations such as customized specific photoactivation and photoconversion efficiencies for convertible fluorophores that allow for sequential activation [57] and tailored imaging buffers for selected organic dyes.

Even more complex imaging experiments involve additional considerations, such as the need to carefully choose the spectral overlap between donor emission and acceptor excitation for optimal FRET, the selection of appropriate strategies for optimal multiphoton absorbance or when utilizing fluorophores as biosensors [82].

### Labeling strategies

Choosing a strategy to label the biomolecule of interest is a crucial part of the experiment. Luckily, strategies suitable for many biological applications are commercially available and, for the best results, experiments should be planned with the labeling strategy in mind from the very beginning. It is important to emphasize that it is always the label attached to the molecule of interest that is visualized, not the molecule itself—the signal we see on the microscope is a label’s length away. Using large labels in combination with high resolutions can thus lead to artificial inflation of the structure [4, 5]. The size and dipole orientation of the fluorophore and the achievable labeling density directly impact the resolution attained. High density—which requires at least an average nearest-neighbor label distance of less than twice the sampling rate according to the Shannon–Nyquist criterion—is necessary [124], or important sample information can be missed. Here, we evaluate the common strategies used (see also Table 3).

**Table 3:**
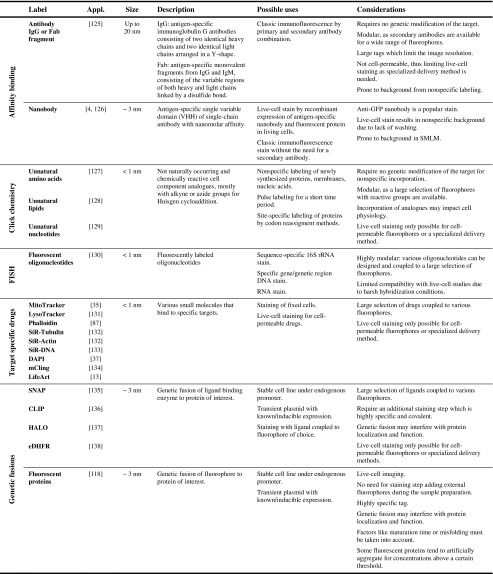
Summary of labeling strategies commonly used in super-resolution microscopy

Affinity-based labeling is probably the approach most widely used across all fluorescence microscopy applications [104, 105, 125, 139]. Antibodies can target virtually any cellular component as an antigen, making the technique extremely flexible. Using combinations of primary and secondary antibodies also makes the approach very modular. Nevertheless, the technique suffers from several drawbacks. First, background due to nonspecific staining is quite common [140], and antibodies may detach from their targets when irradiated with high laser intensities [141]. Second, a typical primary and secondary antibody combination is ∼20 nm in size, which is sufficient to cause imaging artifacts at resolutions realized in super-resolution microscopy. Nanobodies [4], ∼3 nm single-variable domains of single-chain antibodies isolated from cameloids, virtually eliminate this size problem. They can also be fused to fluorescent proteins and recombinantly expressed in live cells [126]. Aptamers—small RNA structures that function much like antibodies and are suitable for live-cell staining [142]—are worth mentioning, though their use is currently limited by poor availability. Much promise is shown by the GFP mimic family of aptamers, which form a GFP-like chromophore when bound to a nonfluorescent substrate [143].

Click chemistry is the term used to describe a set of reactions that occur at high yields in aqueous environments under mild conditions. It thus allows for effective labeling of biomolecules based on the incorporation of unnatural analogues of amino acids [127], nucleotides [129], or lipids [128] carrying a reactive chemical group such as an alkyne, azide, or cyclooctene into cellular structures. Fluorophores carrying the complementary group can then be covalently bound via (for example) cycloaddition [129]. Live-cell imaging is possible with some modifications [144]. This method is suitable for imaging DNA, RNA, proteins, and membranes, and produces very low background fluorescence but usually does not target specific biomolecules. Genetically programmable site-specific unnatural amino acid incorporation can be realized by codon reassignment [145, 146].

Fluorescence in situ hybridization (FISH) [130] allows nucleic acids to be labeled by complementary oligonucleotide probes coupled to fluorophores. The technique is often employed with 16S rRNA complementary probes to study microbe communities [147]. In super-resolution applications, it is a powerful tool for studying chromatin structure and organization, gene location [148, 149], RNA localization and quantification [148], telomere structure [150], etc. As the hybridization protocols involve harsh chemical and temperature treatments, this technique has limited live-cell compatibility.

Engineered ligand-binding enzymes which are genetically fused to the protein of interest are the basis of protein tags such as SNAP [135], CLIP [136], HALO [137], and eDHFR [138]. Such an enzyme label can then be stained by covalently binding its fluorophore-bound specific ligand (benzylguanine, benzylcytosine, chloroalkanes, and trimethoprim, respectively). Such ligands can be fused to virtually any fluorophore, which makes these tags very popular in multicolor applications [66, 95, 106].

Specific labeling options are available for several targets. Fluorescently labeled phalloidin is a toxin commonly used as a filamentous actin stain [87]. SiR-actin, SiR-tubulin [132], and LifeAct [13] are live-cell cytoskeleton stains. Some fluorescently labeled lipid analogues [151] and the recently developed mCling peptide [134] have been used as direct membrane stains. Other target-specific drugs include organelle specific probes such as the mitochondrion stain MitoTracker [35], the lysosome stain LysoTracker, or the ER stain ER-Tracker [131].

All these methods require the introduction of an extrinsic fluorophore into the cell. In fixed cells, this is usually not an issue, and this process can be greatly facilitated by introducing a permeabilization step in which the cell membrane or wall is perforated. Live-cell applications necessitate the use of membrane-permeable fluorophores such as the rhodamine dyes SiR [101], TMR-STAR [106], and Oregon Green [66] and, to a lesser degree, some oxazine dyes such as ATTO 655 [95]. Membrane permeability can be improved by performing certain modifications such as fusion to a permeable peptide [152]. Many alternative strategies for fluorophore delivery, such as electroporation, bead loading, membrane transfer, and micro- or nanoinjection techniques have been developed over the years [153–157].

The discovery and subsequent cloning of green fluorescent protein (GFP) [158] introduced the possibility of small, endogenous, and inherently fluorescent labels. Fluorescent protein fusions, which require no further staining, have become a widespread labeling strategy and are available in a variety of colors [118]. They are highly suited to live-cell studies as long as the cells are carefully checked for physiology after the genetic modification. Unfortunately, they exhibit relatively poor photostability and at best a fifth of the brightness of organic dyes [159]. Since the resolution achievable in SMLM increases with the square root of the amount of photons emitted by a single fluorophore, this can directly impact the resolution of SMLM [46]. Factors such as protein folding as well as the efficiency and velocity of chromophore maturation are important and can differ depending on the environment, e.g., the presence of molecular oxygen is usually needed for final chromophore maturation [160, 161]. Their properties can be readily modified by changing the amino acid sequence, and several versions have been designed to have improved brightness and photostability [162] and switching properties for SMLM imaging. These include photoactivatable proteins such as paGFP [119] and PAmCherry1 [59], reversibly switchable FPs such as Dronpa [120] and Dreiklang [163], and photoconvertible FPs such as Kaede [164], mEos2 [77], or Dendra2 [94]. An often overlooked factor is codon usage bias, and all endogenous tags, including self-labeling enzymes, should be codon-optimized for the organism used [165].

### Controlling the fluorescence of the sample

Robust control of the molecular states is crucial in most super-resolution microscopy applications. Certain steps can be taken to improve the stability, longevity, and intensity of fluorescence, as well as to achieve the on and off switching required for SMLM.

Considering that most microscope cameras record with millisecond-range exposure times, triplet-state transitions and collisional quenching events—which occur several orders of magnitude faster than these exposure times—are not registered as individual events but rather as a loss of signal intensity. Collisional quenching is mostly avoided by imaging in defined, impurity-free buffer solutions, though water shows absorbance in the visible range of light due to its molecular vibrations, as shown in Fig. 3ai. When these molecular vibrations are in resonance with the emission wavelength of a fluorophore, the fluorophore can transfer its excited-state energy to a water molecule during a collision. Heavy-water (D_2_O) molecules vibrate at significantly lower frequencies due to the presence of deuterium. Substituting water in the imaging solution with D_2_O thus increases the overall photon yield. The magnitude of fluorescence enhancement in D_2_O versus H_2_O for a specific fluorophore thus correlates with the spectral overlap of the light absorption of H_2_O with the emission of the fluorophore, as seen for different fluorophores in Fig. 3ai [72].

Molecular oxygen plays an important role in many of the fluorophore’s electronic state transitions. Since the ground state of molecular oxygen is also a triplet, it easily reacts with a triplet-state fluorophore in an electron transfer reaction. This can return the fluorophore to its ground state, but it also produces singlet oxygen and reactive oxygen species (ROS), which can then cause irreversible photobleaching [86]. To avoid the bleaching caused by a buildup of ROS, oxygen can be removed from the imaging solution by adding enzymatic systems such as a combination of glucose oxidase, glucose, and catalase (GLOX) [166], a mix of protocatechuate dioxygenase and protocatechuic acid (PCA/PCD) [167], or a system containing pyranose oxidase, glucose, and catalase [168], as summarized in Table 4. It is worth mentioning that it has recently been reported that most commercial glucose oxidase preparations used in the popular GLOX system suffer from nuclease contamination. Such contamination can cause fluorescent background and introduce artifacts into nucleic acid studies. Furthermore, the GLOX reaction lowers the pH of the solution over time, while other systems do not [177]. Fast, efficient chemical oxygen removal has also been reported to be achieved with methylene blue (MB) and mercaptoethylamine (MEA) [170].

**Table 4:**
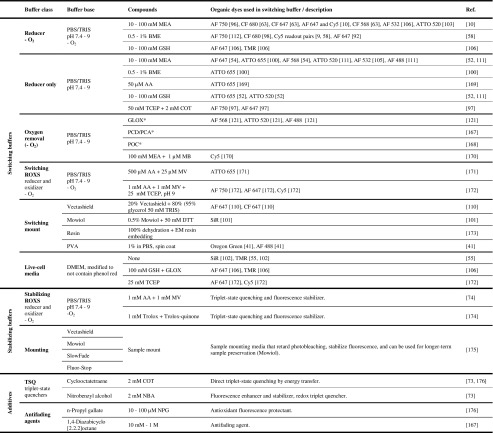
Summary of labeling strategies commonly used in super-resolution microscopy

The switching buffers section of the table includes a list of working dye/buffer combinations. Example buffer compilation: decide on buffer class (e.g., “Reducer with –O_2_”), and then decide on the buffer base, the pH, oxygen removal (−O_2_), and the final compounds based on the fluorophore used (e.g., CF 680 with 10–100 mM MEA [63])

AF Alexa Fluor, –O_2_ oxygen removal, for the abbreviations of the chemicals, see the text or the corresponding references

* For the exact formulations of GLOX, PCD/PCA, and PCO, see the corresponding references

However, since oxygen is such an efficient triplet-state quencher, its depletion can result in a high fraction of fluorophores populating the triplet state, significantly impairing the photon yield. There are several strategies that enable us to circumvent this problem while still removing the risk of bleaching by ROS. The first is to add the chemical cyclooctatetraene (COT), which, much like oxygen, directly returns triplet-state fluorophores to their ground states. As this process significantly shortens the residence time of the fluorophore in the triplet state, the overall fluorescence is stabilized and intensity fluctuations are reduced, as shown in Fig. 3aii [73, 176].

The second approach is to quench the triplet state by colliding the fluorophore with certain reducing agents, thus converting it into the dark, nonfluorescent, anionic radical form F^●−^, as shown in Fig. 3aiii. To do this, chemicals such as mercaptoethylamine (MEA) [176, 178], β-mercaptoethanol (BME) [166], dithiothreitol (DTT) [111], glutathione (GSH) [111], 6-hydroxy-2,5,7,8-tetramethylchroman-2-carboxylic acid (Trolox) [174, 179], ascorbic acid (AA) [74], and potassium iodide (KI) [180] can be added to the imaging buffer. However, to return to the ground state, the F^●−^ fluorophore must be oxidized, a task in which oxygen again plays a crucial role. Removing oxygen can thus lead to very long F^●−^ dark states, a property exploited in SMLM. Using high excitation light intensities ensures that the fluorophores are quickly cycled into the triplet state, from where they are promptly reduced to the dark radical state. Cyanine fluorophores such as Alexa Fluor 647 require a primary thiol (e.g., BME, MEA, GSH, or DTT) in the switching buffer, and undergo a thiol group addition reaction at one of the C atoms in the π-system (Fig. 3cv) [80]. A similar effect can be observed with a phosphine group upon the addition of tris(2-carboxyethyl)phosphine (TCEP) [172]. Since complete oxygen removal is impossible, the few residual oxygen molecules can stochastically oxidize individual fluorophores into the ground state, causing the on and off fluorescence “blinking” desired in SMLM. Many fluorophores develop a distinct absorption peak at shorter wavelengths in their radical state, likely due to disruption of the π-system. Indeed, in cyanine dyes, this system is thought to be practically split in two [80]. Irradiation by UV light thus expedites the return of F^●−^ fluorophores to the ground state [75], as depicted in Fig. 3aiv. Embedding samples in resin greatly suppresses collisional interactions, meaning that reactivation by UV is the only means of returning to the ground state [64], making the method viable for correlative light electron microscopy [181].

Oxygen in the solution can also be replaced with an alternative oxidizer such as methylviologen (MV) [74] or Trolox, which can be converted into an oxidizing quinone form upon UV irradiation [174]. The blinking rate can be adjusted by fine-tuning the ratios of these compounds while keeping the reducer at a high concentration and the oxidizer at a low concentration [171]. The exact concentrations heavily depend on the redox potentials of the fluorophore–reducer/oxidizer pairs. Since the reduction potential of the F^●−^ state varies with the fluorophore considered, different fluorophores can exhibit different blinking behaviors in the same buffer. The pH influences the redox potentials of the compounds in the solution, so changing the pH provides yet another way of adjusting blinking rates [111]. For some fluorophores, the reducing environment inside living cells is sufficient to induce long dark states [106]. In some cases, oxygen removal can be omitted and the addition of a reducer is sufficient [169].

In the cases of STED and SIM, the same strategy can be used to stabilize the fluorescence. Provided the concentrations of both the reducer and the oxidizer are high enough (usually in the millimolar range), the triplet state is efficiently reduced to a radical state that is rapidly oxidized back to the ground state upon formation. The rapidity of this process of reduction and oxidation significantly shortens the overall time the fluorophore spends in nonfluorescing states. The stabilizing effects of a reducing and oxidizing system (ROXS) such as that shown in Fig. 3aiii were reported before the development of SMLM [74].

Fluorophores can also be protected from bleaching by the addition of antioxidants such as* n*-propyl gallate (nPG) [176] or antifading reagents such as nitrobenzyl alcohol (NBA), paraphenylenediamine (PPD), 1,4-diazabicyclo[2.2.2]octane (DABCO), and commercial products such as Vectashield, Fluor-stop, Mowiol, or SlowFade [175, 182]. Vectashield has also been reported to be an effective and very simple SMLM switching medium for several dyes, acting through an unspecified mechanism [110]. COT, NBA, and Trolox have also been (covalently) linked to fluorophores, introducing the concept of “self-healing” dyes [183]. All of these components as well as some popular SMLM buffer formulations that facilitate photoswitching of common fluorophores are summarized in Table 4.

In addition to electronic state transitions, switching is also caused by conformational changes in the chromophore or its surrounding environment. Three main conformational blinking mechanisms exist in fluorescent proteins: photoactivation, photoconversion, and photoswitching [184]. In photoactivatable fluorescent proteins such as paGFP, interactions between the chromophore and a side chain in the beta-barrel stabilize the conjugated π-system in a neutral nonfluorescent state. Irradiation with UV decarboxylates the side chain, shifting the equilibrium of the chromophore towards its anionic state, thus making the protein fluorescent (Fig. 3ci) [76]. Similarly, photoconvertible fluorescent proteins such as mEos2 [77] undergo a fluorescence wavelength shift from green to orange when a peptide bond in the chromophore is cleaved by UV irradiation, causing an extension of the π-system, as seen in Fig. 3cii [185]. Finally, photoswitchable fluorescent proteins undergo reversible on and off switching as a result of UV-induced* cis*/*trans* isomerization like that shown for the fluorescent protein Dronpa in Fig. 3ciii. The isomerization causes protonation changes similar to those that occur in photoactivatable proteins, but which result in the reversible formation of a nonfluorescent form of the fluorophore [78]. Switching properties of fluorescent proteins can be adjusted by modifying the amino acid sequence. Two interesting examples of this are IrisFP and NijiFP [116], which can both be irreversibly photoconverted by UV light from their initial green fluorescing form into an orange fluorescing form, as well as reversibly photoswitched between their fluorescing and dark state (in both the green and the orange fluorescent forms).

Organic dyes can also be made nonfluorescent by inducing reversible changes to the molecule through either* cis*/*trans* isomerization [186] and the addition of certain chemical groups [187] or reduction by NaBH_4_ [188] in a process called photocaging, as seen in Fig. 3civ for a rhodamine dye [189]. Irradiation with the correct wavelength returns the molecule to its fluorescent state [79, 189, 190]. Slow stochastic activation followed by prompt bleaching enables the use of such dyes in SMLM [79, 190].

The novel dye HMSiR represents a class all of its own. This silicon-rhodamine-derived dye naturally resides in a nonfluorescent cyclized form (Fig. 3cvi). It very rarely undergoes a spontaneous change in conformation and becomes fluorescent for a short time. Since this blinking does not require a specialized and probably live-cell-incompatible buffer and is independent of the excitation light intensity, it is very suitable for live-cell imaging [81].

The photochemical properties of individual fluorophores are especially important when designing multicolor experiments. Some fluorescent proteins need specific conditions for proper folding or switching. PAmCherry, for example, requires oxygen for activation, so it cannot be used in oxygen-free buffers [53, 161]. Further, the optimal imaging conditions of a fluorophore partially depend on its redox potential; a buffer that induces blinking in one fluorophore may stabilize another. Table 2 covers most of the working dual- and triple-fluorophore combinations used in SMLM to date.

## Super-resolved cell biology

**Fig. 4a–b:**
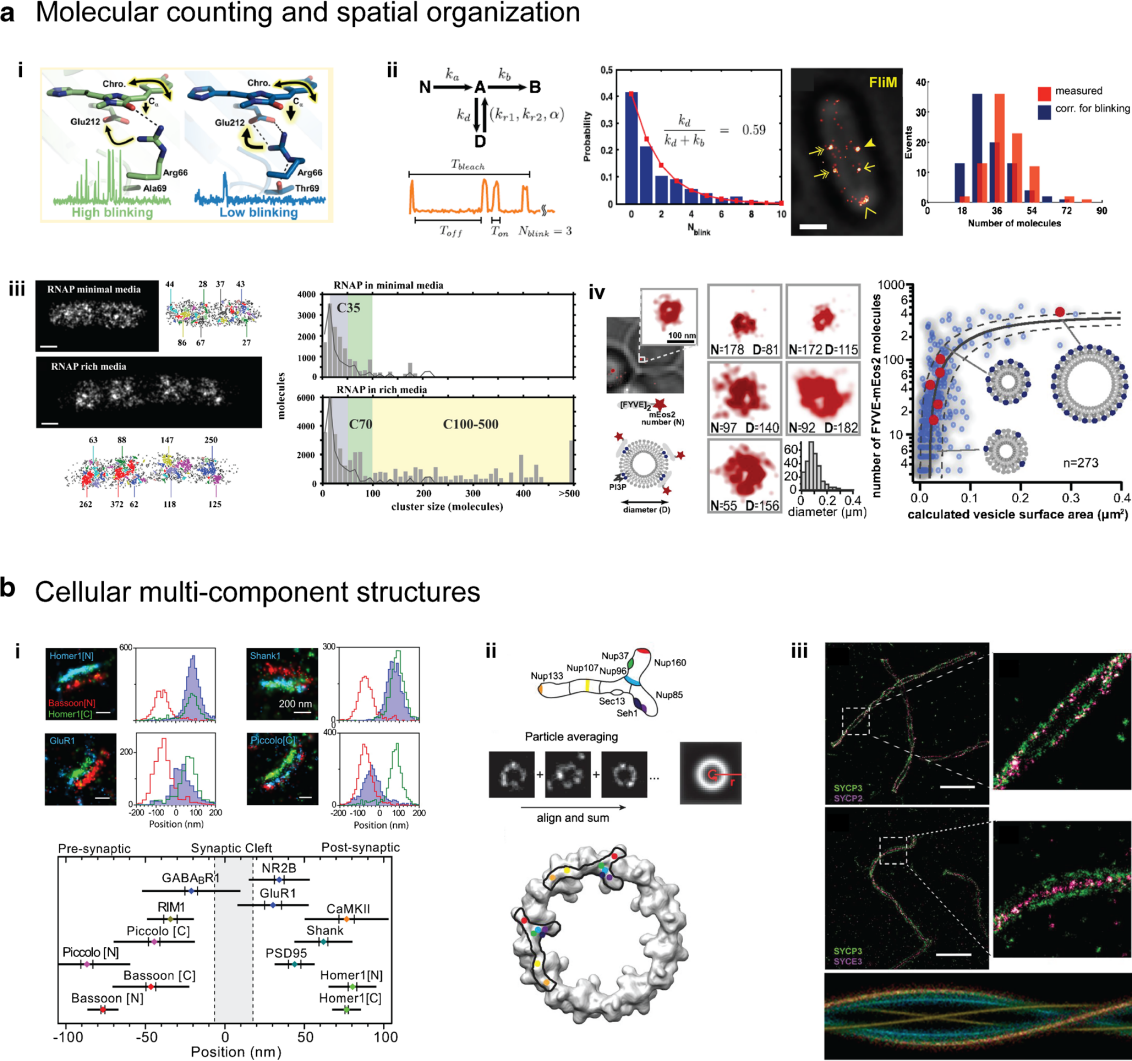
Quantitative super-resolution microscopy. **a** SMLM allows the stoichiometry of a molecule to be determined, with several over- or undercounting effects taken into account. (*i*) The photochemical properties of fluorescent proteins lead to specific blinking and bleaching behaviors. The high-blinking and fast-bleaching behaviors shown by mEos2 (*left*) and Dendra2 (*right*), respectively, are largely determined by the orientation of the single residue arginine 66. Reprinted with permission from [191]. (*ii*) Fluorophore blinking behavior can be corrected for using kinetic fluorophore schemes. In this strategy, the number of FliM proteins per flagellar motor is counted in vivo. Reprinted from [192]. (*iii*) Spatial organization of *E. coli* RNA polymerases under minimal as well as rich growth conditions. Reprinted with permission from [193]. (*iv*) Maturation of endocytic vesicles into late endosomes. Reprinted from [194]. (*b*) Structural super-resolution microscopy reveals the molecular architecture of cellular multicomponent complexes. (*i*) Mutual organization of various pre- and postsynaptic proteins in relation to the proteins Bassoon and Homer1. Reprinted with permission from [139]. (*ii*) Combining data from identical particles yields a high-resolution average. Systematic SMLM imaging of the Y-shaped subunit of the nuclear pore complex allows it to be aligned onto the electron density of the nuclear pore (*bottom*). Reprinted with permission from [104]. (*iii*) Aligning different pairs of synaptonemal proteins onto a helical template yields the three-dimensional model of the synaptonemal complex with isotropic resolution. Reprinted from [105]. Scale bars: **a** *ii* and **a** *iii* 500 nm; **a** *iv* 100 nm; **b** *i* 200 nm; **b** *iii* 2 μm

Direct observations of the molecular processes that take place in cells can help to advance our understanding of life and how the complex interdependencies of single molecules enable it. Using super-resolution microscopy, we can follow these molecules, measure diffusion and progress in assembly processes, and quantify the molecules in subcellular structures at unrivaled spatiotemporal resolution. Over the past decade, rapid developments in these techniques have created a wide spectrum of advanced experimental settings that have already unraveled several mysteries associated with cells, some of which are depicted in Fig. 4 and are briefly summarized below.

### Molecular counting and spatial organization

SMLM data is built on individual single-molecule localizations, and thus allows the absolute stoichiometry of molecules in subcellular structures to be determined.

Here, several effects which compromise this straightforward strategy must be considered. First, undercounting of molecules occurs when some molecules are not counted during the experiment due to, for example, incomplete labeling by the fluorophore, immature or misfolded genetic fluorescent tags, limited photoactivation or switching efficiencies, or insufficient algorithmic registration. The latter problem can be resolved to some degree by using multiemitter fitting algorithms or fluorophore density estimators when there are high fluorescent spot densities, thus decreasing the number of missed localizations [43]. Second, overcounting effects can occur when localized local background fluctuations lead to falsely included positions, during multiple target counting by multiple antibodies or multiple fluorescent labels, or due to multiple localizations per fluorophore caused by blinking behavior. Uncorrected SMLM data can thus result in apparent self-clustering of localizations, which tends to be misinterpreted as clustered organization of the target molecule. These effects can be reduced by, for example, using a fast-maturing one-to-one endogenous fluorescent protein fusion with (engineered) low-blinking behavior. Recently, a mechanistic study revealed that the side-chain conformation of arginine 66 seen in Fig. 4ai is sufficient to cause the popular fluorophores mEos2 (left) and Dendra2 (right) to either blink or bleach, respectively. Consequently, the engineered single mutants mEos2-A69T and Dendra2-T69A show completely swapped behaviors [191].

To further account for miscounting effects, SMLM localizations can be tracked for fluorescent emissions spanning several imaging frames, weighted by known fluorophore detection efficiencies, and statistically corrected for fluorophore blinking [192, 194–196]. For example, as seen in Fig. 4aii, Lee et al. introduced kinetic fluorophore models and accessed the blinking probability of the fluorescent protein tag in order to then count 33 molecules of FliM protein per bacterial flagellar motor in vivo [192]. Further, the varying spatial organization of DNA transcribing *E. coli* RNA polymerase for different metabolic conditions (Fig. 4aiii) as well as the maturation pathway of small endocytic vesicles which form at the membrane and then develop into late endosomes in yeast (Fig. 4aiv) have been revealed.

### Cellular multicomponent structures

Using super-resolution methods, not only can individual molecules be precisely localized, but the large molecular architecture of multiprotein complexes and whole organelles as well as the organization of the genome or membrane can be targeted in the native cellular environment. This yields detailed quantitative molecular maps that capture these large assemblies and place hundreds of different molecules into assembled three-dimensional structures while maintaining the high spatiotemporal resolution of the method. These structures, such as the synapse depicted in Fig. 4bi, can allow us to advance our molecular understanding of their functions and reveal large-scale cellular organization. In the study shown in Fig. 4bi, various pre- and postsynaptic proteins were imaged in relation to the N-terminal localization of the protein Bassoon and the C-terminus of Homer1 by triple-color SMLM, which elucidated their mutual organization in proximity to the synaptic cleft. Indeed, the macromolecular assemblies studied so far using super-resolution techniques comprise an impressive list, including the nuclear pore complex, the ESCRT transport machinery, the neuronal architecture, focal adhesions, the centrosome and cellular division, the endocytosis pathway, as well as the organization of chromatin and membrane lipid domains (see the detailed review in [197]).

Averaging the data for super-resolved identical particles involves combining the individual copies of the same structure into a high-resolution average that complements the single images. This is useful because a single image may have some information missing due to absent affinity labels, imperfect photoswitching of the fluorophores, or nonisotropic resolution. Such a particle averaging strategy can help to elucidate the compositions and organization of macromolecular structures; for instance, the organization of the Y-shaped nuclear pore complex (NPC) subunit Nup107-160 was retrieved and matched with the electron density of the cytoplasmic ring of the nuclear pore via systematic two-dimensional SMLM imaging (Fig. 4bii) [104]. Aligning two-color and two-dimensional SMLM data from different pairs of synaptonemal proteins onto a helical template yielded a three-dimensional model of the synaptonemal complex with isotropic resolution (Fig. 4biii) [105]. The centrosomes of* Drosophila* [198], yeast [199], and humans [200] were studied by combining three-dimensional SIM images. Thus, super-resolution microscopy combined with particle averaging complements current structural biology studies, as it can target structures that are too large for cryoelectron microscopy or when preparation for X-ray crystallography fails. In this context, techniques like subtomogram averaging [201] adapted for three-dimensional super-resolution microscopy could allow us to resolve even more structures at higher in situ resolution, and correlative interaction networks combining super-resolved data with other (e.g., biochemical or genetic) analyzes could lead to large systems biology approaches, which could further refine current studies.

### Live-cell imaging

**Fig. 5a–c:**
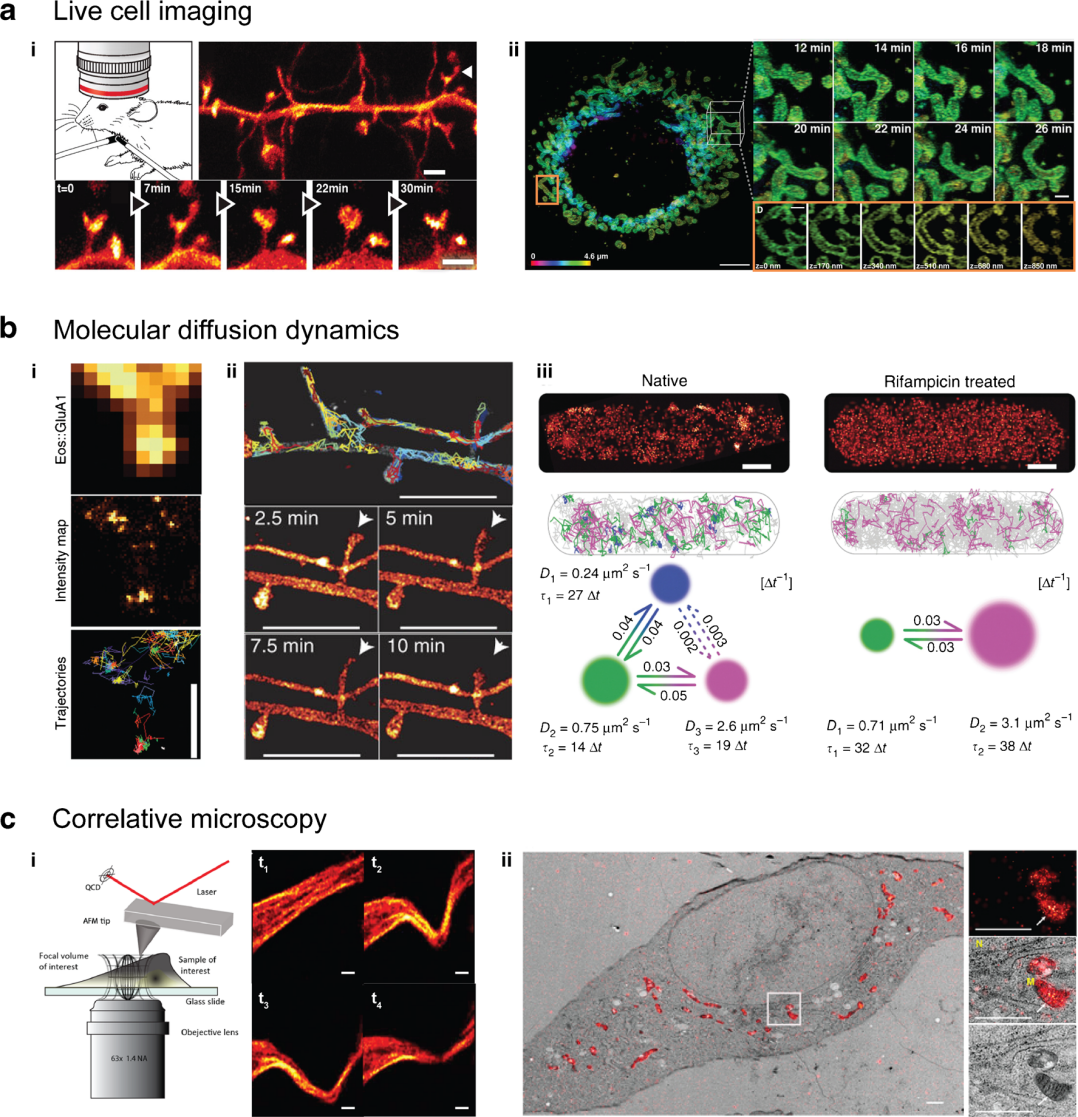
Advanced dynamic and correlative super-resolution microscopy approaches. **a** Live imaging has been successfully performed on living cells and mammals. (*i*) STED microscopy of the dynamics of dendritic spines (*arrows*) in the visual cortex of living, YFP-transgenic, anesthetized mouse. Reprinted with permission from [203]. (*ii*) Mitochondrial fusion and fission dynamics imaged over a period of several tens of minutes by nonlinear SIM in lattice light sheet configuration. Reprinted with permission from [13]. **b** Single-particle tracking schemes elucidate molecular diffusional dynamics. (*i*) High-density tracking of AMPA receptors reveals confined nanodomains in the postsynaptic regions. Reprinted with permission from [204]. (*ii*) In contrast, membrane-bound GPI demonstrates a more homogeneous diffusion. Reprinted with permission from [4]. (*iii*) Bayesian hidden Markov model assessment of Hfq protein dynamics in *E. coli* cells. When mRNA synthesis is inhibited, the fraction of Hfq-binding mRNA (state of slowest diffusion) disappears. Reprinted with permission from [205]. **c** Correlative microscopy allows diverse features of a sample to be measured. (*i*) STED microscopy combined with atomic force microscopy (AFM) visualizes the response of the cytoskeleton upon nanomanipulation by the AFM tip. Reprinted with permission from [206]. (*ii*) Correlative PALM and electron microscopy of the mitochondrially targeted fluorescent protein mEos4 verifies its intact photoconversion and fluorescence under heavy osmium tetroxide fixation. Reprinted by permission from [114]. Scales: **a** *i* 1 μm; **a** *ii* 5 μm (*left*) and 1 μm (*right*); **b** *i* 800 nm; **b** *ii* 2 μm; **b** *iii* 500 nm; **c** *i* 2 μm, **c** *ii* 1 μm

Fluorescence microscopy plays a key role in revealing the structures and functions of living cells in a minimally invasive manner through the use of genetic tags, and thus profits greatly from efficient genome engineering, such as the developing CRISPR-Cas technique [202]. For example, by applying STED microscopy to the visual cortex of YFP-transgenic and anesthetized (but live) mice, it has been possible to observe fine details and measure the dynamics of the tiny dendritic spines in vivo (Fig. 5ai) [203]. In order to improve live-cell super-resolution microscopy strategies, new designs are mainly focusing on three goals: accelerating the imaging speed, lowering the phototoxicity, and expanding the field of view in the lateral as well as vertical directions, all without compromising the resolution. The most critical issues to address are the laser intensities and imaging times used, which, depending on the wavelength of the laser and the irradiation dose, can compromise the health of the cells being studied [141, 207, 208]. Possible solutions involve developing new fast switching fluorophores that can be applied at lower laser irradiations; fluorescent proteins that are usable in the longer (less toxic) near-infrared wavelength range, thus permitting deep-tissue imaging; as well as protected dyes that are shielded from the environment, similar to a fluorescent protein barrel. Such a protective structure could then also shield the cell from ROS and free radicals originating from photochemical reactions in the proximity of the chromophore during excitational or switching processes. New technical implementations which optimize the use of the limited photon budget per fluorophore as well as the imaging speed are favored, such as methods which confine but also parallelize the imaging by multifocal or lattice-like excitation or allow for multifocal detection [209–212]. New rapid and large sCMOS detectors increase the observation volume and allow for faster SMLM switching [3].

During the last few years it has therefore become possible to image whole living cells and organisms over longer timescales. One recent implementation of various types of SIM designs is a three-dimensional nonlinear SIM developed by Li et al. that uses photoswitchable fluorophores combined with lattice-lightsheet microscopy to show endocytic and cytoskeletal dynamics as well as the fission and fusion of mitochondria of whole live cells by labeling their membranes. They resolved the intracellular dynamics at individual mitochondrial constrictions over a period of several tens of minutes at a resolution of about 45 nm laterally and 170 nm vertically (Fig. 5aii) [13].

Another technical advance that is currently being explored is the implementation of adaptive optics to correct for aberrations in large sample volumes, e.g., multicellular organisms [213]. All three-dimensional localization microscopy algorithms have been challenged to participate in a large assessment, in order to evaluate their performance and identify common limitations on them. The results should ultimately guide development work aimed at optimizing three-dimensional SMLM resolution when studying protein ultrastructure in vivo [214].

### Following molecule diffusion dynamics

Single-particle tracking schemes directly monitor the kinetics of intracellular processes. In combination with photoactivatable fluorescent proteins in sptPALM, it is possible to follow the diffusion of individual proteins on cell membranes as well as in the cytoplasm of living cells, and to measure thousands of short single-molecule trajectories by sequential photoactivation. High-density tracking of the GluA1 subunits of AMPA receptors in the membranes of dendritic spines of live hippocampal neurons has revealed their discrete organization in 70–100 nm diameter nanodomains, and has shown that the receptors are mainly immobilized in the postsynapse (Fig. 5bi) [204], in contrast to the membrane-anchored glycosylphosphatidylinositol-GFP (Fig. 5bii). It is also possible to classify individual trajectories by their apparent diffusion constant into distinct states of diffusion, corresponding, for example, to different states of binding. This allows spatial and temporal heterogeneities in protein properties to be resolved. These are normally hidden in ensemble averages but are highly valuable when used in mathematical models for systems biology: by performing Bayesian assessments of hidden Markov models that combine the information from all short trajectories, it has recently been shown that the diffusion constants and state transition rates as well as the number of states in the model can be extracted [205]. This approach has been tested for the protein Hfq in *E. coli*, which mediates post-transcriptional gene regulation by facilitating interactions between mRNA and noncoding small RNA. The Hfq dynamics are highly altered when transcription is blocked using the drug rifampicin, as this decreases the mRNA level in the cell. The state with the slowest diffusion—most likely Hfq binding to the mRNA being transcribed—disappears, and the fraction occupying the intermediate state decreases substantially (Fig. 5biii).

### Correlative microscopy

Correlative microscopy combines the advantages of and opportunities provided by different methods, and thus allows different features of the exact same sample to be measured. For instance, a correlative approach can combine dynamic tracking studies with structural imaging when mapping intracellular vesicle transport on the cytoskeleton [71]. Also possible is the real-time visualization of the fast responses of the cytoskeleton of HeLa cells upon physical nanomanipulation by an atomic force microscopy tip in correlative STED microscopy (Fig. 5ci) [206]. Another direction in correlative imaging is to combine the high ultrastructural resolution and cellular context information of electron microscopy with the specific localization of molecules in super-resolution microscopy (Fig. 5cii) [215]. Unfortunately, most current correlative schemes still suffer from complex and tedious fixation protocols as well as limited labeling and imaging strategies, i.e., the cryo- or resin-covered environments used for electron microscopy impair the photophysics of most standard fluorophores. However, technical implementations develop rapidly; for instance, a correlative fluorescent protein tag, mEos4, which fluoresces and photoconverts normally under heavy osmium tetroxide fixation has recently been developed (Fig. 5c, ii) [114], and the dye TMR has been shown to not only preserve its fluorescence during high-pressure freezing and freeze substitution preparations, but to be able to photooxidize diaminobenzidine (DAB) too, which then yields high electron-microscopic contrast [173, 216].

In an optimistic but still realistic future, super-resolution microscopy will push beyond its current limits of routinely achieving experimental resolutions of tens of nanometers to approach the distances of typical single-molecule FRET measurements (2–10 nm) as well as the structural resolution of cryoelectron microscopy and X-ray crystallography (about 2–3 Å), which will allow us to more directly combine the heterogeneity and dynamics of protein complexes measured in vivo with the precise structural information available from purified protein complexes.

## Outlook

Over the last few decades, super-resolution microscopy has proven its value in the life sciences, and a myriad of biological applications of super-resolution microscopy have emerged. The super-resolution toolbox currently consists of many diverse methods and application strategies that complement traditional cell biology studies as well as techniques from molecular biology or biochemistry. Super-resolution microscopy is on its way to becoming a standard research tool, which is leading to a huge demand for computer-based data processing and openly available analysis software for (advanced) data evaluation, visualization, and comparison, as well as accessible, affordable, and simple-to-use hardware implementations. Also, super-resolution microscopy traditionally yields large volumes of microscopic data, which would ideally be stored and handled in open-access public platforms. Whether this vision comes to pass largely depends on the development of new algorithms as well as open-source software and strategies for efficient large-scale data handling.
